# An enactive account of placebo effects

**DOI:** 10.1007/s10539-017-9572-4

**Published:** 2017-04-12

**Authors:** Giulio Ongaro, Dave Ward

**Affiliations:** 1 Department of Anthropology, London School of Economics, Houghton Street, London, WC2A 2AE UK; 2 Department of Philosophy, University of Edinburgh, Dugald Stewart Building, 3 Charles Street, Edinburgh, EH8 9AD UK

**Keywords:** Affordances, Embodied cognition, Enactivism, Placebo effects

## Abstract

Placebos are commonly defined as ineffective treatments. They are treatments that lack a known mechanism linking their properties to the properties of the condition on which treatment aims to intervene. Given this, the fact that placebos can have substantial therapeutic effects looks puzzling. The puzzle, we argue, arises from the relationship placebos present between culturally meaningful entities (such as treatments or therapies), our intentional relationship to the environment (such as implicit or explicit beliefs about a treatment’s healing powers) and bodily effects (placebo responses). How can a mere attitude toward a treatment result in appropriate bodily changes? We argue that an ‘enactive’ conception of cognition accommodates and renders intelligible the phenomenon of placebo effects. Enactivism depicts an organism’s adaptive bodily processes, its intentional directedness, and the meaningful properties of its environment as co-emergent aspects of a single dynamic system. In doing so it provides an account of the interrelations between mind, body and world that demystifies placebo effects.

## Introduction

In a now famous study, Moseley et al. (2002) performed ‘sham arthroscopic surgery’ on a group of subjects with chronic osteoarthritis of the knee. Moseley et al. found that most patients receiving the sham surgery, where anaesthetic was administered and incisions made to the knee, but no arthroscope was inserted and no rinsing or scraping occurred, recovered knee functions at the same rate of patients receiving the real surgery. For some patients, this ‘intervention’, bogus by the standards of medical science, did more to ease chronic and debilitating symptoms than years of standard treatment and therapy.^1^

This is a striking example of a placebo effect. Roughly, a placebo is simply a treatment that isn’t supposed to have therapeutic effects on the condition being treated; placebo effects are thus surprising beneficial effects that run counter to this supposition. Correlatively, *nocebo* effects are negative effects of treatments that are supposed to be inefficacious.^2^ In recent years many clinical trials, meta-analyses, neuroimaging studies and experiments have considerably expanded our knowledge of such effects, showing them to be pervasive and at times powerful. We know, for example, that placebo effects are elicited in a wide variety of diseases, ranging from conditions of pain to some diseases of the nervous system such as Parkinson’s or migraine; from gastrointestinal and genitourinary dysfunctions to disorders of the immune and endocrine systems (Benedetti 2014). In a series of experiments, the neuroscientist Fabrizio Benedetti and his team found that even the most powerful modern painkillers are substantially less effective when administered surreptitiously through intravenous injection than when administered in the patient’s full view (Benedetti et al. 2011). And all but the most recent history of medicine consists primarily in the history of the placebo effect. Most of the treatments predating the development of modern European medicine, whether in Greece, India or China, are seen by modern eyes as ineffectual—at times bizarre—placebos (Majno 1975; Shapiro and Shapiro 1997)

The prevalence and surprising power of placebos looks puzzling for science-based medicine. Here, we aim to show that this puzzlement stems from a particular way of thinking (firmly and understandably entrenched in western culture) about the mind’s relation to the body and the world, and a corollary view of the nature of medical science and treatment (Lee 2012). We will argue that an alternative ‘enactive’ conception of the mind/body/world relation (Varela et al. 1991; Di Paolo 2005; Thompson 2007) can accommodate and elucidate the phenomenon of placebo effects.^3^

## The puzzle about placebos

Since the Enlightenment, science has had great success conceiving of and explaining observable phenomena in broadly mechanistic terms. We live in a physical universe, governed by determinable laws. All observable phenomena are products of particular configurations of matter unfolding in law-governed ways. If we want to understand, predict or control some phenomenon, we need to identify the physical conditions that give rise to it, and the laws that determine the way it unfolds.

Among the areas where this paradigm has succeeded most impressively is medical science. Just like the rest of the universe, we can predict, understand, and sometimes control, our physical ailments by identifying the physical conditions that underpin them, and the laws that govern their unfolding. Here, the parts of the universe most relevant for understanding and treating disease are our bodies. Medical science becomes the study of human bodies and their constituent mechanisms and functional constants. Following the rest of physical science, it operates by reducing the area of study to progressively smaller units and sub-units, and searching for universal laws and structures of linear causality between different bodily mechanisms.

This framework provides medicine with a natural criterion for defining the pathological and the normal. A trait or function is pathological to the extent that it varies from the mean of its measurement within a given population (Boorse 1977, 1997). Disease is simply a quantitative aberration (an insufficiency or exaggeration of some sort) of the normal. Having objectified disease in this way, the role of medical treatment becomes that of restoring a specific abnormal function or trait to its normal state, and its therapeutic effects are scientifically established (via clinical trials) according to its capacity to do so (Canguilhem 1989, 2012). In practice, this means that the analysis of pathology and cure is carried out through localization, tackling disease at the levels of the organ, tissue, cell and gene, depending on the disease in question. Like a repairman, the doctor’s job is that of fixing a mechanical problem.

Placebo effects mystify because they violate current biomedical understanding of active factors in treatment. In the double-blind randomized clinical trials typical of contemporary medical research, placebo groups are supposed to work as controls to groups receiving the real treatment, because they involve no intervention on the mechanisms hypothesized to underpin the disease. Given this, the fact that placebos can lead to substantial therapeutic effects is puzzling. Medical anthropologist Daniel Moerman suggests an apparently analogous example. Suppose that:

we fabricate some placebo socket wrenches. They look like socket wrenches, sound like them, feel like them. But we design them so that when you put the socket over the loose nut and tighten it, the nut will stay loose. We secretly place these wrenches in the toolboxes of a randomly selected set of mechanics at the car repair shop. Now, if we discovered that the nuts these mechanics were working on really *did* tighten up, we would have good reason to be surprised. The only thing that can tighten up nuts is a (real) wrench. (Moerman 2002, p.137).

In clinical trials, placebo groups receive a treatment selected in virtue of the apparent lack of any mechanism linking its effects to the bodily condition requiring treatment. Against this backdrop, the fact that placebos often have substantial bodily effects looks as mysterious as the effects of Moerman’s placebo wrenches. If we understand disease in terms of deviations from the statistically normal functioning of the body’s processes, and its treatment in terms of interventions upon the body’s mechanical workings that aim to restore normal functioning, interventions that result in healing without targeting those underlying mechanics are puzzling.

Of course, placebos *do* appear to target something—the patient’s *beliefs* about whether they are undergoing treatment and thus their expectations about whether they will get better. But how, in cases of placebo effects, can a mere belief be causally efficacious? Someone’s false, but sincerely held, belief that their severed limb will grow back, or that their broken bone is perfectly intact, neither makes their limb less severed, nor their bone less broken. So why should a false, but sincerely held, belief that one has had arthroscopic surgery, or that one has been given a causally efficacious pill, bring about bodily changes that constitute healing? The puzzle about placebos consists in reconciling a mechanistic conception of the body and nervous system (and a corollary conception of disease and health) with the evidence that the patient’s belief in the efficacy of their treatment has a causal effect on their body, even when this belief is false. From a mechanistic perspective, the only difference between the recipients of Moseley’s placebo surgery and a control group who receive no treatment at all is the presence of a false belief on the part of the former group. But how can the mere presence of a false belief have such dramatic effects?

## Problems with existing accounts

This puzzle, we will argue, doesn’t arise for enactivists. Enactivism provides an alternative conception of disease and health, and of the relationship between mind and body, that renders placebo effects unmysterious. Before making our case for this in the next sections we briefly consider the problems faced by existing accounts of placebo effects. We do this firstly to show that there is a live puzzle about how to understand placebo effects, and secondly because—as we shall see—each of the problematic accounts below contains important insight.

### Conditioning

Early attempts to accommodate and explain placebo effects appealed to classical conditioning (Wickramasekera 1980; Ader 1999; Siegel 2002). Past histories with objectively neutral objects such as doctors, hospitals, pills and injections, condition us to respond in ways beneficial to healing when in the presence of those objects. Thus, being given a pill or an injection with no apparent efficacy for the symptoms being treated can nonetheless produce a healing response.

However, it seems that placebo effects can occur without conditioning—for example, the patients in Moseley et al.’s sham surgery did not require prior exposure to surgery in order to exhibit their marked improvements. There are also studies suggesting that expectations about the effects of treatment are, in some cases, more important determinants of placebo effects than prior conditioning. Benedetti et al. (2003) gave subjects a saline injection after informing them that the injection would increase the levels of pain they felt—thus inducing an *expectation* of *increased* pain. One trial group, however, had undergone prior conditioning using an analgesic, intended to forge a *conditioned association* between the injection and a *reduction* in pain. Despite this, both groups in the trial reported an increase in the pain they experienced, suggesting that expectation was a more powerful determinant of the effect than prior conditioning.^4^

### Expectations

Partly in response to such problems, *expectancy theorists* have reinterpreted conditioning in cognitive terms (Rescorla 1988; Kihlstrom 2002). For these theorists, conditioned learning about placebos is not directly dependent on the pairing between the conditioned and unconditioned stimuli, but on the information contained in the controlled stimulus (Kirsch 1997; Stewart-Williams and Podd 2004). We have, then, the addition of a cognitive variable. This is called ‘expectancy’, and is taken, in existing accounts, to be a consciously accessible representation of a physiological outcome, which the expectancy is supposed to trigger. Thus, receiving an injection from a medical professional that we are told will increase our levels of pain will have this effect because we expect injections to have the effects that our doctors say they will.

One source of problems for expectancy theorists is evidence suggesting that conditioned responses to stimuli *do* play a role in producing placebo effects. For example, Benedetti et al. (1999) administered the opioid buprenorphine to a group of patients, producing respiratory depression as a normal side effect. After treating patients with buprenorphine a few times they gave them a placebo, telling them it was the same pain medication. Shortly after administering the placebo, they measured significant respiratory depression. Patients did not form any conscious expectancy about this particular physiological response—when asked, patients claimed that they had not noticed the respiratory depression, suggesting that they lacked expectations about it.^5^

Partly inspired by such worries, Haug (2011) suggests that placebo effects are mediated not by conscious expectations, but by *aliefs*. In addition to the empirical problems for expectancy theories, Haug argues that any cognitive states mediating placebo effects have content that need not be consciously accessible, and that can be inferentially isolated from the contents of the subject’s beliefs. Moreover, states mediating placebo effects must, it seems, be able to directly cause bodily changes. This suggests that such states are very different from expectations standardly construed as beliefs about likely consequences. The content of our beliefs, it is standardly thought, can usually be brought to conscious awareness, should stand in appropriate inferential relations to the contents of our other beliefs, and can cause bodily changes (i.e. behaviour) only in conjunction with another propositional attitude such as a desire. Haug thus suggests that we should understand placebo effects as mediated by *aliefs*, as defined by Gendler (2008a, b). Among other properties, aliefs are stipulated to directly cause behaviour, and have contents that are associatively rather than rationally related. This makes them more plausible candidate states for mediating placebo effects than expectations as standardly construed.

We agree with Haug’s diagnosis of the problems faced by the expectancy theorists. But the proposed explanation in terms of aliefs is, as it stands, unhelpful. The lesson of the failures of conditioning and expectancy theories is that a unified account of placebo responses must do justice to the appearance that they occupy a middle ground between conditioned response and the fulfillment of an expectation. The contribution of an alief-based account is to posit a class of states lying between automatic physiological responses to stimuli and the inferentially-related, reflectively accessible states posited by orthodox belief/desire psychology. But in this context the appeal to aliefs just gives a name to the domain we’re puzzling over, rather than explaining its existence. It still needs to be made intelligible why an alief about healing should bring about the state of affairs that specifies its content. My expecting Elvis to enter the room does nothing to bring that physical state of affairs about—so why should my expecting particular changes in my body bring that state about? Likewise, if alieving that Elvis is about to enter the room does nothing to bring that about, why should things be different in the case of my aliefs about my bodily states? Saying that aliefs can directly cause bodily changes amounts to stipulating that things *are* different in the latter case, but without making intelligible how or why this could be.

### Evolutionary accounts

Finally, some theorists attempt to explain placebo effects in terms of hypotheses about their evolutionary function. For example, Humphrey (2002) and Humphrey and Skoyles (2012) suggests that placebo effects occur when the inhibition of a latent capacity for self-healing is overridden as a result of perceiving oneself to be in an environment that affords healing. Fully mobilizing the body’s immune resources has a high metabolic cost, so Humphrey hypothesizes an evolved ‘health governor’, capable of determining whether these resources should be deployed based on the perceived context. For example, it’s usually better to deploy one’s immune response in the context of treatment caringly offered by other people than when fleeing a hungry lion. Thus, we have evolved an automatic brake on our self-healing capacity that is released only in appropriate contexts. Building on Humphrey’s general framework, Evans (2003, 2005) suggests that placebos trigger a suppression of an ‘acute-phase’ immune response via the release of endorphins, and a subsequent reduction in pain and inflammation. Finally, Steinkopf (2015) argues that some conspicuous symptoms of immune response (such as pain, swelling and fever) can signal the need for treatment and care from helpful bystanders, and might have evolved partly for this reason. His hypothesis is thus that once these symptoms are perceived to have performed their signaling function—for example when one perceives oneself as undergoing treatment—they will diminish, regardless of the other effects of the treatment.

We find each of these proposals about the adaptive value of placebo responses interesting and plausible, and the above suggestions about the biological underpinnings of placebo responses might form an important part of a completed account. However, as they stand, each of these accounts faces problems. For example, there are many placebo-responsive conditions (such as depression, Parkinsons and epilepsy) whose biophysiological manifestations appear unrelated to the acute-phase or inflammatory responses emphasized above by Evans. The diversity of placebo responses makes it hard to see how a single evolved biological mechanism could explain them all. Most important, for our purposes, is a residual puzzle concerning how healing processes are triggered by appropriate contexts. Not all placebo responses require the presence of caregivers, and the range of contexts associated with a healing response is much wider (and often unrecognizably more sophisticated) than those available to our evolutionary ancestors. As we saw above, the relationship between treatment context and placebo response can neither be a matter of simple conditioning, nor of explicit expectation. An account in terms of *implicit* expectations (such as Haug’s) owes us a story of the content of these expectations, how they arise, and how they relate to the biological dynamics of placebo responses. This is what our enactive account aims to provide.

### The enactive alternative

Each account above contains important insight. The enactivist agrees with the evolutionary theorist that placebo responses are grounded in biological processes, and that they occur in virtue of experiencing oneself as in a context that affords healing. They agree with conditioning theorists that placebo responses need not be mediated by beliefs about the treatment, and that they are a function of the subject’s history of environmental interactions. But they also agree with expectancy theorists that placebo responses are dependent, in a way to be specified, on the subject’s attitude toward the treatment.

According to the account we will provide here, placebos afford healing by shifting attention from mal-adapted parts of our bodies to (an expanded range of) things we can achieve with them; they do so in virtue of the culturally inculcated meanings we perceive in placebos and their contexts. This can happen because the parts of our bodies, our overall bodily relationship to the environment, and the practically and culturally meaningful structures within that environment, are all co-emergent and co-dependent aspects of a single dynamic process. The sections that follow briefly outline the distinctive and interrelated ways in which enactivists understand the relationships between: an organism, its parts, and the environment (“Autopoietic organisation and minimal normativity” Section); sensorimotor activity and cognition (“Adaptivity, cognition and valence” Section); adaptive activity and intentionality (“Enactive attention” Section); affect, intersubjectivity, and cognition (“Cognition is affective”, “Cognition is intersubjective” Sections). Considering these features of enactivism lets us see, in turn: the distinctive conception of disease and healing available to the enactivist (“An enactive conception of disease and healing” Section); how the enactivist can make sense of a bodily attentional dimension to illness and placebo effects (“Somatic attention, health and placebos” Section); and how enactivism can accommodate important findings about cultural determinants of placebo effects (“Cognition is intersubjective” Section). We aim not to provide arguments that compel assent to these features of enactivism and their theoretical consequences, only to set out the relevant features of an enactivist framework in sufficient detail to show that such a framework provides a way of making sense of a pervasive and puzzling empirical phenomenon.

## Organism, environment and cognition

### Autopoietic organisation and minimal normativity

Perhaps the most important guiding idea of enactivism is that when we are studying a living being (or its mind) we are studying an *organism*, whose organisational principles and properties differ in fundamental ways from those of a machine. For enactivists, the defining feature of living organisms is their ‘autopoietic’ organisation. Consider a single cell. It is separated from its chemical environment by a semi-permeable boundary, and a network of metabolic processes both regulates the internal constitution of the cell and exchanges matter with its surroundings (nutrients go in, waste products go out). These processes simultaneously knit the cell into its environment and render it distinct, creating and sustaining the boundary that marks it out as an organism. Thompson (2007) summarises:

Thus the cell embodies a circular process of self-generation: thanks to its metabolic network, it continually replaces the components that are being destroyed, including the membrane, and thus continually re-creates the difference between itself and everything else. (p.99)

Living organisms thus interact with their environment in ways that create and sustain their status as distinct biological entities.

For our purposes here, two properties of this autopoietic organisation merit special attention. Firstly, the reciprocal, co-productive relationship between organisms, their constituent parts and processes, and their environments. The metabolic processes that constitute an organism consist in the activities of its component parts—but those parts are themselves generated and sustained by the functioning of the organism which encompasses them. The autopoietic organisation of the organism renders it distinct from its environment—but this organisation consists in a dynamic network of processes that is indifferent to that distinction, spanning the organism/environment boundary whilst at the same time creating and sustaining it. So, for the enactivist, the parts of an organism, the organism itself, and the environment with which the organism interacts emerge simultaneously from a shared dynamic tangle of processes. It is thus essential to living organisms that they create and sustain their constituent parts and processes, and that their doing so involves a reciprocal causal relationship with their environment.^6^

Secondly, note that this organisational structure of living organisms entails that their functioning entails a kind of minimal normativity—in constituting itself as an autopoietic unity by creating and sustaining a distinction between itself and its environment, an organism supplies a standard by which we can evaluate the functioning of its parts and processes. Qua autopoietic unity, insofar as an organism’s functioning allows it to persist as an entity distinct from its environment in the face of external perturbations we may say it’s doing *well,* or *succeeding*; insofar as its functioning fails to sustain its distinction from the environment (as when a bacterium dissolves into the molecular soup that surrounds it) we may say it’s doing *badly,* or has *failed*. Without the autopoietic organisation that allows an organism to stand out from its environment these particular standards could not be applied—there would be nothing to which we could apply them.^7^

### Adaptivity, cognition and valence

We might worry (with Di Paolo 2005) that the ‘normativity’ which autopoietic organisation allows us to impute to an organism’s functioning is of limited use in grounding an account of cognitive capacities. As defined above, autopoietic organisation admits of no degrees—an organism’s constituent processes either succeed in sustaining it as an entity distinct from its environment, or they don’t. According to contemporary enactivists (Di Paolo 2005; Thompson 2007),^8^ understanding the biological roots of cognition requires focusing instead on the related concept of *adaptivity.* Adaptivity is here defined as an organism’s capacity to regulate its relationship to the environment (and hereby regulate its internal states—recall the dynamic tangle above) in ways that help keep itself around as an autopoietic unity. Unlike autopoiesis, this organisational property of organisms *does* permit assessments of their functioning that admit of degrees. When an organism regulates its functioning in a way that moves it further away from its conditions of viability, we can say it becomes *better* adapted to its situation. It is this property of organisms that endows features of an organism’s environment, and of its embodiment, with *significance* for its viability.

Enactivists often illustrate this with the example of a bacterium in a sugar gradient (Varela 1997; Di Paolo 2005; Thompson 2007). Because of the physical constitution of a bacterium, sucrose-rich environments help keep its functioning within the parameters required for it to persist as an autopoietic unity. And because of the details of its embodiment, the sensorimotor dynamics of its relationship with its environment ensure it will tend to move away from portions of its environment with low concentrations of sucrose, towards sucrose-rich areas. According to enactivists, this sensorimotor capacity of an organism to modulate its relationship to the environment in viability-sustaining ways is the basis of all cognition. The relationship between sucrose and the bacterium’s sensorimotor dynamics and conditions of viability lets us say that sucrose has *significance*, or *value* from the bacterial perspective, and that the bacterium’s path up the sugar-gradient *aims at* the sucrose-rich area.

### An enactive conception of disease and healing

Above, we suggested that placebo effects currently appear mysterious against a backdrop of a mechanistic conception of bodies, and a corollary conception of pathology as statistical deviation of body parts or processes from their mean within the relevant population. Enactivism helps demystify placebo effects in part by suggesting an alternative to this *quantitative* conception of pathology. To see how, let’s consider a couple of problems for this conception.

Firstly, we can note (with Canguilhem 1989) that it neglects what appears to be an essential *qualitative* aspect of disease and health. Statistical deviance looks plausible as a definition of the pathological only insofar as we usually experience it in negative terms—for example of lack, or constraint. In cases where this isn’t so, we are rightly suspicious of the quantitative definition. If, for example, large numbers of deaf or autistic people lucidly judge that their lives are perfectly happy and fulfilled with respect to the standards they endorse, we have reason to question whether they are rightly understood as suffering from an illness or pathology, or as requiring healing. An enactivist framework naturally accommodates this insight. We saw above that, for the enactivist, understanding an organism as composed of essentially distinct parts or components is importantly distortive. Organisms are self-sustaining unities, dynamically coupled to their environments. Focusing on the statistical propriety of a trait, function, or body-part in isolation (as the quantitative conception invites us to do) thus entails losing sight of the facts that make it the case that we are dealing with an aspect of an organism. As we have just seen, enactivists are particularly interested in *adaptive* organisms—those that regulate their relationship with the environment to keep their functioning within bounds of viability. And it is this capacity that allows features of an organism’s environment, its activity, and its embodiment to have meaning or significance from the organism’s perspective. This suggests a natural explanation of what, for the enactivist, is misleading about the quantitative conception—in abstracting away from the role a body-part plays in the dynamic process that constitutes and sustains the organism as a unity in a meaningful environment, we remove the resources required to make sense of the essential qualitative aspect of disease and health. For enactivists, the possibility of things going well or badly from an organism’s perspective is grounded in the dynamic co-constitution of organism, parts, and environment. Consequently, understanding a body part in abstraction from these dynamics involves losing sight of the properties that make it potentially relevant to the health and flourishing of the organism.

Secondly, Goldstein (1939) makes the related point that medical conditions are not pathological ‘in themselves’, but in virtue of the ways in which they close down possibilities for adaptive interactions with the environment. Cancer, for example, is a disease not because of the statistically abnormal patterns of cellular growth it entails, but because of the effects those patterns can have on an organism’s relationship with its environment. As before, enactivists can naturally accommodate this insight. We saw in “Adaptivity, cognition and valence” Section how enactivist cognition is rooted in an organism’s capacity to modulate its relationship to its environment in ways that sustain its conditions of viability. Pathology, then, can be understood as any condition that affects the dynamic relationship between organism and environment in a way that moves the organism away from its optimal conditions of viability. Healing can be understood as any modulation of that relationship that moves the organism towards those optimal conditions.^9^ Again, enactivists diverge from the quantitative conception by insisting that body parts and processes must be understood as constituents of an adaptive dynamic process in order to understand their significance for an organism’s flourishing. One important consequence of this view is that the possibility of healing and pathology arises when, and only when, the dynamics constitutive of a living cognitive system are in place. Since cognition consists in the regulation of organismic dynamics in viability-promoting ways, healing itself is simply an instance of the dynamics fundamental to life and mind.

Enactivists can thus sum up the problems with the quantitative conception as follows: In understanding disease as the statistically deviant functioning of some body part or process, and healing as the restoration of statistical propriety, the quantitative conception understands our bodies and their constituent parts and processes mechanistically. But this obscures the very conditions of possibility for sickness and health. Living organisms, not machines, are kinds of things that can be literally ill, or healed, and enactive accounts of cognition take our status as living organisms as their starting point. This entails stressing the autopoietic and adaptive relations between environments, bodies and their parts, and the way in which these relations create a perspective from which properties of the organism’s activity and environment have significance. The contrast between the quantitative and the enactivist conceptions of health stems from the enactivist claim that adaptive dynamics of living systems bring with them the possibility of applying normative standards to the functioning of the organism, and thus understanding its constituent parts and processes as bearing on its flourishing, or otherwise. This is why enactivists can accommodate the qualitative dimension of illness in a way the quantitative conception cannot.

The next sections will show how enactivists can build on the material outlined so far to construct a framework within which placebo effects appear unmysterious. But we can already glimpse how enactivism might afford an account of the relationship between body, environment and cognition that does this. Enactivists claim that we can understand the relevance of body parts or processes to the health of an organism only by emphasising their dynamic entanglement with the organism as autopoietic unity, which is in turn coupled to its environment. In “The puzzle about placebos” Section we saw that placebo effects look mysterious because they arise from interventions that are designed to lack causal efficacy on the afflicted body part or process. But if an organism, its parts, its environment and its cognitive life are entwined in the tight web of interrelations the enactivist describes, then touching one part of this web will make the rest reverberate. Tinkering with the environmental portion of this web, or with the cognitive processes the web realises, will have bodily effects. And this makes it intelligible how manipulating cognition or the environment could be a way of healing the body.^10^

## Adaptivity, attention and intentionality

### Enactive attention

Recall (from “The enactive alternative” Section) that we are working towards an account according to which placebos afford healing by shifting attention from mal-adapted parts of our bodies to (an expanded range of) things we can achieve with them. ‘Attention’ here refers just to the way in which the organism is intentionally directed toward features or properties within its environment. We already hinted (in “Adaptivity, cognition and valence” Section) that enactivists hold that the most basic form of intentional directedness—what Merleau-Ponty (2012) calls ‘motor intentionality’—arises from an organism’s adaptive engagement with its environment. The dynamics of the bacterium’s interaction with its environment bring it about that particular properties and structures (such as the presence or absence of sucrose) have significance for it, and allow us to say that the bacterium’s activity is directed towards particular aspects of its environment. Similarly, enactivists hold that the dynamics of our interactions with the environment bring it about that we are intentionally directed towards properties and structures within it, and that those properties and structures have significance for us as solicitations or impediments to particular activities or projects. The first important point about the conception of attention required for our purposes, then, is that it is conceived of as a mode of intentional directedness toward the environment that arises from the dynamics of embodied sensorimotor interactions. Attention here is an *active* and bodily, or *somatic*, relation between organism and environment.^11^

We can bring the second important point into focus by noting that enactivists—again, following Merleau-Ponty—hold that this directedness always has a figure/ground structure. Our activity is always directed towards its object through another process that can itself become the object of attention. Consider, for example, how our experience is directed when engaged in skillful hammering. When all goes well, one’s attention is directed through one’s activity towards the outcome at which it aims. We don’t experience ourselves as manipulating a hammer, but as acting on a nail. But things can break down—the head flies off the hammer, involuntarily shifting attention to the bodily interaction with the hammer that formerly served as the ground of our attention to our task; a muscle cramps, involuntarily shifting attention to bodily movements that served as the ground of our manipulations of the hammer. Another example: imagine holding your hand against a kettle that is gradually coming to the boil. Initially, your experience might be directed towards the tactile and temperature properties of the kettle. But a point comes where your attention involuntarily shifts towards a property of your hand, as the kettle becomes too hot. Bodily properties and processes that previously functioned as the *ground* of your intentional directedness towards the environment now become the *figure*—the object of your awareness. Your experience of this gestalt shift involves phenomenal continuity; the property of your hand on which your attention is now focused is not experienced as recently and suddenly instantiated, but as something that previously occupied the background of your awareness that has, as a result of a gradual change, shifted to the foreground. The second important point, then, is that the bodily relationship to the environment characteristic of attention functions as the ground against the backdrop of which the organism can be intentionally directed towards distal objects.

The final important point is that we should not construe attending to some property or feature as turning additional cognitive resources toward a feature of the world or our awareness that was already there. Attending changes and helps constitute its object. As Merleau-Ponty puts it:

To pay attention is not merely to further clarify some pre-existing givens; rather it is to realize in them a new articulation by taking them as *figures*. They are only pre-formed as *horizons*, they truly constitute new regions in the total world. The original structure that they introduce is precisely what makes the identity of the object before and after the act of attention appear […] attention is the active constitution of a new object that develops and thematizes what was until then only offered as an indeterminate horizon. (2012, p.32–3)

This is an extension of a point from “Autopoietic organisation and minimal normativity” Section, where we saw that features and properties to which an organism’s activity is geared are constituted by bodily interactions between organism and environment—they are enacted. In the above kettle example, whilst there is continuity to our phenomenology, there is also change; our new mode of attention helps constitute the pain in our hand as an object of awareness.

Momentarily, we will consider how this conception of the links between attention and bodily activity can add to our understanding of placebo effects. First, we should briefly clarify our attitude towards a tenet of enactivism that we have handled delicately thus far. Standardly (e.g. Weber & Varela 2002; Di Paolo 2005; Thompson 2007), enactivists endorse a thesis of *strong continuity* with respect to the relationship between life and mind, claiming that the processes fundamental to mindedness are merely suitably enriched instances of the processes fundamental to life. Hence, they claim, bacterial cognition is of a kind with our own, since each arises from the same sensorimotor dynamic properties. Moreover, most enactivists (following Jonas 1982; Weber and Varela 2002) think that this involves *phenomenological* continuity—the simple adaptive dynamics that characterize a bacterium’s relationship to its environment support a simple mode of awareness that differs in complexity, but not necessarily in kind, from our own. We have tried to present our account in a way that remains neutral with respect to these claims. In “Adaptivity, cognition and valence” Section, for example, we spoke of adaptive dynamics affording *us observers* a standard according to which organisms can be evaluated as doing well or badly, and features of their environment can be accorded significance. Standardly, enactivists would hold that the relevant dynamics do more than afford us a way of ascribing such features to the organism/environment relationship; adaptivity entails that those features really obtain, that they are immanent in the functioning of the organism. Moreover, these features have a phenomenological manifestation, however primitive, for the organism. At least one of us has reservations about whether such claims of strong continuity are entailed by the enactivist apparatus presented so far (see e.g. Villalobos and Ward 2015; Ward and Villalobos 2016). However, the enactivist account of placebo effects does not depend on strong continuity. Setting simpler organisms aside, as long as it is the case that for *us humans* adaptive interactions with our environment embody a form of motor intentional directedness and involve a phenomenological component, we have the necessary materials for our enactive account of placebo effects. Giving a full defense of these enactivist claims is beyond our scope here—but so long as they are in fact defensible, we have what we need for our account.^12^

### Somatic attention, health and placebos

How does this active, somatic conception of attention help us understand placebo effects? We saw above (“An enactive conception of disease and healing” Section) that enactivism suggests conceiving of health in terms how body parts and processes are integrated into adaptive organism/environment dynamics. The above remarks on attention allow us to expand on this. Borrowing an elegant quote from Leriche, Canguilhem states that “health is life lived in the silence of the organs” (1989, p. 102). In health, attention is directed towards solicitations and impediments in the environment through bodily functioning; the bodily relation to the world functions as the ground against which opportunities and obstacles show up. But this can be disrupted. The dynamics characteristic of smooth, unreflective coping can break down, and attention can be correspondingly redirected toward aspects of bodily functioning that now show up as impediments to a goal, rather than the ground against which that goal appears. This is what happens when our attention is drawn from the nail we are hammering to the cramp in our arm, or from the temperature of the kettle to the pain in our hand. A natural conception of health for the enactivist, then, is in terms of the dynamics characteristic of an optimal relationship to the environment for an organism;^13^ a natural conception of illness is in terms of disruption of those dynamics, such that an organism’s activity becomes directed toward its own bodily states or processes, rather than the distal properties to which those states and processes usually attune it. We also saw (in “Adaptivity, cognition and valence” Section) that a system’s capacity to modulate its bodily relationship to the environment in ways that move it toward conditions of optimality (*adaptivity*) is the hallmark of a cognitive system. This allows the enactivist to conceive of healing as a process that is simultaneously bodily and cognitive, and fundamental to cognitive systems.

What does this suggest about placebo effects? We now have a conception of health in terms of an organism/environment relationship that allows the organism’s awareness to be directed toward distal properties and features in a way conducive to its flourishing, and a corollary conception of illness in terms of a breakdown of this relationship and the resulting direction of awareness towards bodily properties and processes that impedes optimal interaction with the environment. As noted in “An enactive conception of disease and healing” Section, conceiving of health and illness in terms of the dynamic web of organism/environment relations makes it intelligible how intervening on the environment could be a way of healing the body. We can now add that the relevant interventions will be those that manipulate the organism/environment dynamics in ways that provoke exogenous shifts of attention from the body (and the ways it impedes adaptive interactions) to the environment (and the ways in which it affords adaptive interactions). For enactivists, then, placebos can be understood as environmental structures or processes that *afford healing* through their capacity to shift attention in this way.^14^ Think, for example, of a hobbled distance runner finding herself unexpectedly capable of a sprint finish when she rounds a corner and encounters the finish line and cheering friends. A nocebo has the converse effect, prompting suffering or discomfort through drawing attention to properties of the body; as when cuts or grazes become more painful once one looks at them. Importantly, the above conception of attention as involving active constitution of the object of awareness suggests that such attentional shifts restructure our intentional relation to the object of awareness. Having attention shifted from the properties of the hot kettle to the properties of one’s hand partly constitutes the pain in one’s hand as an object of awareness. Likewise, having attention shifted from bodily properties to the properties of the environment one is in bodily interaction with can change the intentional object of awareness.

The enactivist account as we have sketched it thus far makes it intelligible why shifts in attention can alter the way in which we are aware of a body part or process—can alter its status as an *intentional* object. But many placebo effects are striking precisely in virtue of alterations in *physical* states. The efficacy of placebo ultrasound in reducing pain and inflammation (e.g. Hashish et al. 1988) is surprising chiefly in virtue of the physical changes brought about by the sham treatment, rather than the change in awareness of the swollen area. There is a residual puzzle, then, about the relationship between intentional and physical changes. Here we can note that the enactivist grounds an organism’s intentional relation to its environment (including parts of its body) in terms of the dynamic web of interactions described in “Organism, environment and cognition” Section—thus intentional changes will coincide with changes in the physical states implicated in those dynamics. A full explanation of the relationship between intentional and physical changes would, however, require a completed story of the dynamics of cognition and how they are implicated in the body and its relation to the environment. Our more modest aim here is to show how enactivism’s general conception of the dynamic relation between mind, body and world renders placebo effects intelligible as a phenomenon, and provides a useful framework in which to investigate them. It does so, as we are now in a position to see, by revealing them as continuous with other unmysterious phenomena with which they share an intentional structure. Consider these cases:

- Olivia has to attend an important reception party but cannot afford the particular designer dress she thinks would be appropriate for the occasion. She feels anxious, uncomfortable, self-conscious about her own presentability, and unable to confidently engage with the other guests. She experiences her body as a problematic object.
- Oliver is a Parkinson patient. His body, which previously allowed him to engage fluently with the world, now offers him resistance, experienced as fatigue and stiffness.

Olivia and Oliver are each prevented from optimally interacting with the world by the ways in which their bodies figure as objects of awareness. Suppose a windfall allows Olivia to buy the dress she thought appropriate. We would not be surprised to learn that this alleviated her anxiety (and its physiological manifestations) and allowed her to interact fluently and confidently with the other guests. Moreover, we would expect it to do so not by occupying Olivia’s attention during the function, but by functioning in the background of her awareness, changing her bodily comportment towards the situation in a way that allows opportunities for fluent interactions to show up for her. Whilst there is certainly much of philosophical interest about this case—some of which we will discuss in the next section—the way in which the dress alleviates Olivia’s anxiety doesn’t raise the kind of puzzles sketched in “The puzzle about placebos” Section. According to the enactivist, the efficacy of placebo treatments upon Parkinson symptoms (Benedetti 2014; Goetz et al. 2008) receives analogous analysis. Providing Oliver with a placebo pill or surgery places him in a new environmental context that he understands as affording healing, and in doing so shifts his attention from maladaptive properties of his body to opportunities for fluent interaction with his environment. In each of these cases we tinker with the environmental portion of organism/environment dynamics to alter the distribution of attention between body and world. And in each case the dynamic links between environment, attention and bodily processes make it intelligible that these environmental changes can have bodily results.^15^

## Some flesh on the bones: affectivity, intersubjectivity and enculturation

These accounts of Olivia and Oliver raise a couple of pressing questions. Firstly, are they really analogous? We suggested that in each case ‘healing’ occurs through altering an agent’s implicit grasp of their practical relation to their situation. But it might be noted that Olivia’s anxiety is an *emotional* state, and that it’s thus intuitively clear how this grasp can alter the physiological manifestations of her anxiety; plausibly, one’s emotional state constitutively depends on such a grasp (Frijda 1987; Lambie and Marcel 2002). However, symptoms such as Parkinson’s tremors or limitations in knee function do not appear to be emotional states. So, an objection might go, the relationship between Olivia’s cognitive and physiological changes looks unmysterious simply because both pertain to her emotional state; this doesn’t seem to be the case with physiological changes associated with Parkinson’s and many other placebo-responsive conditions, so the attempted demystifying analogy fails.

A second important question concerns our appeal to affordances. Why can placebos heal? Because, we appear to have claimed, they afford healing. Isn’t this like ‘explaining’ how the TARDIS works by saying that it affords time travel? An initial response is to note that we have already made important claims about these affordances—the previous section claimed that they are taken up via altering the balance of attention between body and environment, and noted that the enactivist framework outlined so far implies that such alteration will have bodily effects. But, we should wonder, how do these affordances get there? And how do we recognize them? Our enactivist account is incomplete until we have addressed these questions. To do so, we need to understand why, for enactivists, human cognition is essentially *affective* and *intersubjective*.

### Cognition is affective

Enactivists reject the suggested disanalogy between Olivia and Oliver because they hold that *all* cognition has an affective component (Thompson 2007; Colombetti 2007, 2014). We saw in “Organism, environment and cognition” Section that enactivists argue that the sensorimotor dynamics obtaining between an organism’s embodied activity and its environment suffice for a perspective from which structures and properties can show up as *significant* for the organism. A corollary of this is that the activity constitutive of cognition is never disinterested—since it directs the organism toward features of the environment that bear (either positively or negatively) on its flourishing, it always embodies an implicit evaluation of the organism’s relation to its environment. Moreover, we have seen, this evaluative relationship depends on how the organism is directed towards its environment through bodily states and processes. Enactivists thus think that a particular kind of *embodied appraisal* (Prinz 2004; cf. Colombetti and Thompson 2007; Colombetti 2014) account of emotion is implied by their view, and that cognition is never affectively neutral. They agree with appraisal theories of emotion (Arnold 1960; Scherer et al. 2001) that emotional states depend on a grasp of the way in which one’s situation bears on one’s projects and wellbeing, and with bodily or James–Lange theories of emotion (James 1884) in holding that emotional states depend on awareness of bodily states and processes. As the previous section suggests, these aspects of emotional states are intimately related—we are directed towards our situation and its bearing on our projects and wellbeing only through a background awareness of our bodily states, just as our hammering activity is directed towards its object through a background awareness of our body.

Enactivists thus reject the separability of cognition and affect, since they hold that both are products of the same sensorimotoric directedness toward the environment. The fact that the link between Olivia’s cognitive and bodily states involves emotion therefore does not provide a disanalogy between her and Oliver. Instead, it is the way in which enactivism reveals the structural parallels between the two cases that helps us see placebo responses in Parkinson patients as no more mysterious than the physiological changes brought about by Olivia’s dress. Noting the affective component in Olivia’s story helps demystify the relationship between the cognitive and physiological changes it involves. After all, we know that emotions are not just intra-cranial states, but events involving the whole organism, encompassing multiple visceral and motor processes, and regulatory and communicative networks across nervous, endocrine and immune systems (Damasio 1999; Purves 2004). For enactivists, these interrelations between somatic, affective and cognitive processes characterise all cognition. Altering Oliver’s affective and cognitive relationship to his environment by placing him in a milieu that he takes to afford healing and improved mobility thus also effects somatic changes.

### Cognition is intersubjective

Appreciating the inseparability of cognition and affect sets the stage for the final feature of enactivism we must appreciate to understand placebo effects. One’s affective relationship to the environment is, at least for us humans, not set in stone, but dependent on our history. A situation or object that someone else experiences as affectively neutral might be highly salient for me in virtue of my history of embodied interactions with it—think of the different visceral reactions provoked by the whir of the dentist’s drill, or the smell of warm apple pie. But our embodied interactions with the environment, and the affective and attentional patterns that they bring with them, do not develop in a vacuum; they are always shaped and scaffolded by caregivers and conspecifics, within a particular cultural context. As Thompson (2007) insists, ‘human subjectivity is from the outset intersubjectivity, and no mind is an island’ (p.383). Echoing attachment theory (Bowlby 1988), Noë (2009) illustrates:

A two-way exchange arises between child and mother that provides the setting within which the child develops both physiologically and psychologically. A child learns to restore its own calm or comfort by being calmed or comforted by the mother. The child’s basic physiological processes – burping, for example – are facilitated for the baby by the mother. The caretaker manipulates the child’s posture, raising her into a sitting position, now setting her down in a prone position, either to arouse the child or prepare her for sleep. The caretaker directs the child’s attention to this or that and manipulates things for the child … In a very real sense, the baby-caretaker “dyad” is a unity from which the child only gradually emerges as an individual. (p.50)

In emphasizing the role of these patterns of embodied interaction to psychological development, enactivists emphasize the package of innate capacities that Trevarthen (1979) called ‘primary intersubjectivity’—perceptual sensitivity to facial expressions, eye gaze, gestures and tones of voice, and the ability to coordinate behavior in response to these. During development, capacities to gear behavior to another’s gaze and gesture give rise to capacities to share attention and to have one’s attention directed in particular ways (e.g. Gallagher 2008; Noë 2009; De Jaegher and Di Paolo 2007).^16^ As we grow up, socially scaffolded habits thus develop in the ways particular objects, situations and events solicit attention and affectively engage us. In shaping patterns of sensorimotor interaction with the world in this way, enactivists think, enculturated embodied interactions shape the dynamics determining our intentional, attentional and affective relationship to the world.^17^ These interactions can thus teach us to experience, viscerally and immediately, objects as alluring or repulsive, situations as comforting or threatening, and styles of behavior or comportment as reassuring or unnerving. This plasticity in our habits of embodied interaction, and the attentional and affective concomitants of those habits, is not limited to early childhood—it is a permanent feature of our lives. The ways we distribute attention around features of our world, and the patterns of embodied affective responses those distributions entail, are constantly being tweaked, reinforced and renegotiated.

This feature of the enactivist framework does important work in answering a puzzling question with which we began this section—where do the healing affordances in terms of which we explain placebo effects come from? An affordance is a property of an organism’s environment that obtains in virtue of the relationship between organism and environment^18^—chairs, for example, afford sitting to humans because of the relationship between human anatomy and chair design. In non-placebo cases of treatment and recovery, there is no mystery as to why the healing affordance obtains—we have enough understanding of the relationship between the physical, chemical or biological properties of the treatment and those of the target condition to make this intelligible. Placebo effects are puzzling because of the apparent absence of such intelligibility. This is just to restate “The puzzle about placebos” Section’s puzzle about placebo effects in the language of affordances. We have just seen how, for the enactivist, socially scaffolded habits of embodied interaction simultaneously give rise to patterns of somatic changes and concomitant distributions of attention. These habits, formed through our history of interactions with medical practitioners, treatments and apparatus, explain why the mere presence of an appropriate medical context can be enough to trigger the distribution of attention—away from the body and back towards the environment (Sect. “Somatic attention, health and placebos”)—and corollary somatic changes (Sects. “Enactive attention”, “Somatic attention, health and placebos”, “Cognition is affective”) that are characteristic of healing for the enactivist (Sects. “An enactive conception of disease and healing”, “Somatic attention, health and placebos”). The healing affordances of placebos, then, obtain in virtue of the meaning we perceive them to have in virtue of our history of enculturated embodied interaction, and the fact that being intentionally directed towards a meaningful structure in the environment is a bodily and affective relation that entrains specific somatic and attentional patterns. Oliver’s bodily response to the placebo Parkinson’s treatment is thus explained in the same way as Olivia’s embodied response to wearing the designer dress—both treatment and dress prompt new ways of attending to the world that are dynamically intertwined with specific bodily and affective processes; both can do this in virtue of the culturally inculcated meanings they are perceived to have in virtue of a history of embodied interactions.

There is a wealth of empirical data on the cultural variability and determinants of placebo effects that the enactive account is thus well placed to explain. For example, the facts that branded or expensive placebos are more effective than non-branded or cheap ones (Branthwaite and Cooper 1981; Waber et al. 2008; Espay et al. 2015) look difficult to explain without the enactivist’s focus on the interplay between culturally scaffolded perceptions of meaning and patterns of bodily response. Likewise, the enactivist emphasis on how culturally situated interactions shape the bodily dynamics that are recruited in placebo effects suggests how we might frame explanations of the relationship between the size of the effect and the mode of treatment. For example a meta-analysis of migraine treatment trials has found that placebos administered through injection are on average more powerful than oral placebos (de Craen 2000). The respective culturally mediated perceptions of the power of pills and injections might be invoked by the enactivist to explain these results (see e.g. Payer 1996). Finally, a classic instance of placebo responses to visual meaning is relative to drug colour. Stimulants work better if they are red, orange or yellow, whereas sedatives are most effective if they are blue (e.g. de Craen et al. 1996). The reason for this can be sought in the meanings generally attributed to ‘hot’ and ‘cold’ colours, which commonly induce alertness and tranquility respectively. There is, however, an intriguing exception to this pattern. Cattaneo et al. (1970) showed that for Italian men (but not women) blue placebo pills tend to inhibit sleep rather than promote it. How might this puzzling outlying result be explained? Moerman (2002) suggests that we find an explanation in the distinctive culturally inculcated meaning that blue has for most Italian men—as the colour of *Gli Azzurri,* the national soccer team, the somatic and attentional habits built up around blueness are more likely to be skewed towards arousal than sedation for this particular demographic.^19^

## Conclusion

The puzzle about placebos arises from the relationship they present between culturally meaningful entities (treatments or therapies), our intentional relationship to the environment (implicit or explicit beliefs about a treatment’s healing powers) and bodily effects (placebo responses). How can a mere attitude toward a treatment result in appropriate bodily changes? Enactivism is uniquely placed to address this puzzle because of its insistence on the fact that the parts of our bodies, our overall bodily relationship to the environment, and the practically and culturally meaningful structures within that environment, are all co-emergent and co-dependent aspects of a single web of dynamic relations. If the dynamical underpinnings of mind, body and culture are inseparably intertwined as enactivists suggest, there is no mystery as to why tinkering with cultural and cognitive aspects of this dynamic system should have bodily effects. The previous sections have sketched how, for the enactivist, our cognitive lives emerge from a culturally embedded web of dynamic autopoietic and sensorimotor processes that span brain, body and world to knit us into a meaningful environment. As we develop and navigate the world, with the help of others, stable patterns and trajectories emerge in these dynamics—paths, or tracks, are laid down (Varela et al. 1991)—and some of these stable patterns manifest themselves as bodily affective and attentional habits in our responses to the world. Placebo effects, we have suggested, occur when a perceived property of our environment triggers some such habit in a way that entails a pattern of bodily changes that is characteristic of healing. Placebos thus afford healing by shifting attention from mal-adapted parts of our bodies to (an expanded range of) things we can achieve with them, and do so in virtue of the culturally inculcated meanings we perceive in placebos and their contexts.

Returning to the problematic theories we surveyed in “Problems with existing accounts” Section, we can see how the enactivist view can preserve the merits of each while avoiding their difficulties. Like the evolutionary theorist, enactivists hold that placebo effects must be understood with reference to our status as biological organisms—specifically, as organisms characterized by the adaptive and autonomous dynamics described in “Adaptivity, cognition and valence” Section, and the entanglement of an organism, its parts, and the environment that this implies. But the enactivist is not committed to the existence of a single mechanism underlying all placebo effects, and has a built-in answer to the question of how healing is triggered by the perception of an appropriate context. This answer avoids the problems that face conditioning and expectancy accounts by appealing to an embodied intentional relation to the perceived context that lies between conditioned response and explicit expectation. As we have seen (Sects. 5, 6), enactivists appeal to the way a history of socially scaffolded embodied interactions gives rise to habits of somatic and attentional response that are fundamentally bodily while simultaneously entailing a mode of intentional directedness towards the environment. Like an account in terms of aliefs (Haug 2011), then, enactivists hold that the cognitive relationship to the therapeutic context that explains placebo effects is more than a conditioned response, but less than a fully-fledged (articulable, inferentially promiscuous) belief. But the enactivist framework allows us to say more about just what this relation consists in: it is an embodied mode of intentional directedness towards the environment that encompasses affective and attentional components.^20^ Those components, coupled with an enactive conception of healing (Sects. “An enactive conception of disease and healing”, “Somatic attention, health and placebos”) make it intelligible why the perceived significance of a treatment can result in placebo (or nocebo) effects. Finally, appreciating the role of intersubjectivity and enculturation in establishing the dynamics constitutive of cognition allows the enactivist to accommodate important findings about the cultural determinants of placebo effects (“Cognition is intersubjective” Section), and make sense of the unique interplay between culture, cognition and embodiment that characterizes them.^21^

We should once again be clear about the explanatory scope of our account here. The utility of enactivism for understanding placebo effects stems from its general features as a paradigm for understanding cognition. By linking the understanding of the efficacy of a treatment in terms of socially-scaffolded habits of somatic attention, and by grounding those habits in dynamics encompassing both body and world, enactivism makes it intelligible why an understanding—implicit or explicit—of the efficacy of a treatment or therapeutic context can have substantive effects on one’s body, and on one’s capacities for environmental interaction. We have tried to do just enough here to sketch the features of enactivism required to demystify placebo effects in this way. A full enactivist explanation, however, would have to elucidate the specific bodily and interactive processes underlying the somatic and attentional patterns that our account schematically describes. This is what would be required for an explanation of why specific shifts in somatic attention cause particular bodily changes—i.e. for an account of *particular* placebo effects, rather than an account that aims at rendering these effects intelligible in general. Supplying such an explanation is not something we have attempted here. Part of enactivism’s appeal, though, is its provision of a framework within which these issues can be addressed. It might be, for example, that the biological processes mentioned in “Evolutionary accounts” Section, above—Evans’ (2003, 2005) acute-phase response, Steinkopf’s (2015) immune reaction—make important contributions to the dynamics of many placebo effects. Recent attempts to explain placebo analgesia (Büchel et al. 2014) and ‘hysterical’ or psychogenic sensory and motor symptoms (Edwards et al. 2012) in terms of Bayesian models of neural organization look nicely compatible with the enactive framework here. And finally, existing enactivist work by Barandiaran and Moreno (2006, 2008; following Edelman 1987) might house an explanation of the boundaries of the range of placebo-responsive conditions. They distinguish the Nervous System of the Interior (comprising the neuroendocrine system, limbic system and autonomic nervous system) from a Sensorimotor Nervous System, where the former is responsible for the adaptive control of neural and bioregulatory organization, the latter for adaptive sensorimotor interaction. Nonetheless, these two systems are dynamically coupled and it is the dynamics encompassed by this coupling that, according to Barandiaran and Moreno, demarcate the boundaries of a cognitive system. If such a view were correct, then it would naturally suggest that conditions will be placebo-responsive to the extent to which they are implicated in this set of dynamics.

We hope to pursue these attempts to specify the biological underpinnings of the enactive account in future work. Our claim in this paper, though, is that each of these proposals can only contribute to a demystification of placebo effects insofar as it functions to describe one part of the broader web of dynamic relations between mind, body and world, between affect and cognition, and between self and society that we have described here. Sketching the structure of the dense web of interconnections that obtain between these phenomena lets us see them as co-emergent aspects of a single dynamic system, and thus removes the puzzle about their interrelations that makes placebo effects look mysterious. We thus think that enactivism represents an attractive option for making sense of a pervasive and important phenomenon. Enactivism shows us how to demystify placebo effects, and reveals new avenues for thinking about health and treatment. And it does so by showing us a new and fruitful way of putting mind, body and world back together again.

## Footnotes

1 More recently, Sihvonen et al (2013) demonstrated that sham surgery for degenerative meniscal tears was as effective as genuine arthroscopic surgery. Harris (2016) contains a fresh compilation of “placebo surgery” cases.

2 This rough definition of placebo and nocebo effects suffices for our purposes in the paper. Given their intrinsically oxymoronic nature, providing a more nuanced definition has never proven to be an easy task. Nunn (2009) surveys existing attempts and despairs that: ‘Every way of looking at the placebo construct invites criticism because it doesn’t make sense. A placebo is something effective that is inert. Or something inert that has an effect. That doesn’t make sense of either the word ‘inert’ or the word ‘effective’.’ (1018). Recently, a thorough effort at making sense of these terms on a conceptual level was made by Howick (2017).

3 The label ‘enactivism’ has been applied to a wide variety of views. As we shall see in subsequent sections, the kind of enactivism that houses our explanation of placebo effects is one that emphasizes the interrelations between (i) biological organization and sensorimotor interactions, (ii) sensorimotor interactions and cognition, and (iii) cognition and culturally instituted meaningful structures. Notable attempts to articulate a theory of cognition with this structure can be found in the work of Francisco Varela (Varela et al. 1991; Weber and Varela 2002), Thompson (2007) and di Paolo (2005). Related views that emphasise some, but not all, of these interrelations (such as Hurley 1998; Noë 2004, 2012; Hutto and Myin 2013) are compatible with the treatment of placebo effects we provide here to the extent that they could be developed to include theoretical emphases on these three sets of interrelations. See Ward et al. (in press) for a discussion of different forms of enactivism and their interrelations.

4 See also Montgomery and Kirsch (1997).

5 See also Jensen et al. (2012, 2015) for further studies that problematise expectancy theories, showing that placebo effects can be elicited outside conscious awareness.

6 Note the conception of ‘environment’ implied here—environment and organism are co-defined, such that the properties and structures that make up an organism’s environment are that subset of properties and structures involved in the set of sensorimotor interactions that allow the organism to persist as an autopoietic unity. A consequence of this is that different kinds of organisms live in different environments. As we will see later, enactivists hold that the affective and intersubjective constraints on *human/*environment interactions mean that our shared human environment incorporates an exponentially richer range of meaningful properties and structures than the environments of other organisms, and it is this fact that explains the range and power of placebo effects found in humans. See e.g. Gibson (1979), p.8, for a famous historical antecedent of this conception of the relationship between organism and environment.

7 This brief summary of autopoiesis and its consequences suffices for our purposes here. See Maturana and Varela (1980), Thompson (2007) (ch.5), or Di Paolo (2005) for more. We have been deliberately cagey about the way in which we think autopoiesis implies normative standards—see the concluding paragraph of “Enactive attention” Section, below, for more on this.

8 Both of whom offer their accounts as friendly amendments to the earlier, autopoiesis-centric formulations of (e.g.) Varela (1997), Weber and Varela (2002).

9 See Saborido et al. (2016) for a recent attempt to formulate a naturalistic conception of health and disease in terms of adaptive dynamics. Carel’s (2007) phenomenological account of illness overlaps with the characterization we provide here, but is not committed to the enactivist conception of the dynamics we are in the process of setting out.

10 In addition to the possibility of healing the body through intervening on cognition or environment, the enactive framework sketched so far suggests that healing could also take place through changing the norms of optimal functioning that the organism enacts. We saw above (“Autopoietic organisation and minimal normativity”, “Adaptivity, cognition and valence” Sections) how the dynamics of an organism’s relationship with an environment bring about a perspective from which it can be assessed as ‘doing well’ or ‘doing badly’. On the conception of healing just outlined, if an organism’s functioning is disrupted (say, by the loss of a body part), healing can occur by the organism modifying its active relationship with the environment in ways that move it back towards the original dynamics characteristic of optimal functioning. But healing could also take place via a shift in what counts as optimal functioning for the organism—the change in sensorimotor dynamics entailed by the disruption might change the standards of optimality the organism enacts.

11 We say a little more about the somatic processes on which this relation depends in “Cognition is affective” Section.

12 How might such a defense go? One possibility would be to hold (following McDowell 2007) that skillful sensorimotor engagement with one’s environment entails an intentional relation only when it can be integrated with a capacity for reason-giving explanation. One strategy for arguing that such a relation entails a phenomenological component would be to endorse Fish’s (2009) naïve realism, according to which the qualitative properties of experience are simply properties of the objects to which one is related in experience.

13 Importantly, in our human case there is reason to think that we have some say in the nature of these optimal dynamics, in virtue of our partial capacity to set the goals and projects that we take to be constitutive of our well-being. This capacity is Taylor’s (1985) subject when he writes of our status as ‘self-interpreting animals’, and it is in virtue of this capacity that the additional means of healing sketched in n.8, above, obtains. The ways in which this capacity arises is an important topic which we cannot pursue here—though resources for investigating it are found in Taylor’s work.

14 An important precedent for this aspect of our account is Frenkel’s (2008) phenomenology of placebo effects. Like the enactivist, Frenkel draws on Merleau-Ponty to suggest we should understand placebo responses as a form of motor intentional directedness towards affordances for healing. Our account here is entirely compatible with Frenkel’s treatment. But we think that the enactivist’s appeal to adaptive dynamics that span body and world provides extra explanatory purchase on the intimate relation between mind, body and world characteristic of placebo responses. And, as we will see in the next section, enactivism allows us to make sense of the affective and cultural dimensions that are central to many – perhaps all—placebo effects.

15 The analogy presented above further suggests that placebo effects do not have to rest on an explicitly entertained belief about the reality of treatment in order to work (Olivia’s dress might have its effects regardless of her explicit beliefs about the reality of the social or aesthetic properties that make it appropriate for the reception). Interestingly (and in accordance with our account), recent studies have shown that patients can respond to placebo even when they are told that they are receiving a placebo (Kaptchuk et al 2010; Carvalho et al 2016). The act of treatment provision itself, coupled (in the mentioned studies) with the message that ‘placebos can work’ communicated by physicians, affords possibilities of healing and can shift the patient’s attentional state accordingly.

16 For example, Fuchs and de Jaegher (2009) suggest (echoing Vygotsky 1978) that ‘Originally, pointing is only a simple but incomplete grasping movement directed towards a desired object. The child’s failed reaching may provoke a helping reaction from the caregiver… The individual reaching movement can then turn into a ‘gesture for others’. The meaning of reaching changes and develops into a new possibility for interacting with the (social) world: pointing.’ (480).

17 Again, the enactivist’s emphasis on the priority of shared intersubjectivity in a developmental account of our mature experience is anticipated and partly inspired by Merleau-Ponty (1964, 2010).

18 For discussion and a dissenting view, see Chemero (2003). Our account is, as far as we can tell, neutral with respect to the various proposals about the metaphysics of affordances Chemero surveys.

19 Other relevant examples here include: Moerman’s (2000) work on the cross-cultural variability of placebo responses to high blood pressure, and the possibility of explaining this in terms of Payer’s (1996) anthropological studies of cultural variability in attitudes towards, and diagnosis and treatment of, heart and circulatory diseases; Moerman’s (2013) account of the way a gradual increase in the placebo-responsiveness of depression has tracked the way in which depression and its treatment has been portrayed in popular culture.

20 In fact, one of Gendler’s original characterisations of aliefs (2008b) emphasises their status as affectively-laden states, tied directly to behaviour, which can be the results of socially scaffolded habits—see her discussion of implicit associations. Focusing on these properties of aliefs brings out the compatibility between the enactive approach we present here and a suitably developed alief-based account of placebo effects. The key contribution of the enactive account is to make these properties of affect, bodily interaction and social scaffolding central, and to house them within a conception of the dynamic relation between mind, body and world that renders intelligible their mutual influence in cases of placebo effects. Thanks to an anonymous reviewer for prompting clarification here.

21 In an important recent article Alfano (2015) argues that placebo effects can be caused by one of three mechanisms: conditioning, expectation, or somatic-attentional feedback loops, and that on this basis there is little scientific or clinical utility in viewing ‘placebo effects’ as a unified phenomenon. Instead, individual effects should be studied and theorised in their own terms, and implications for informed consent should be considered in light of the particular mechanism implicated. The enactive account differs in holding that these mechanisms are intertwined: the ‘expectations’ involved in placebo effects are understood in terms of habits of somatic attention, which are most often inculcated by a past history of socially-scaffolded interactions.
